# Supplementary material for: Efficacy of morning versus afternoon aerobic exercise training on reducing metabolic syndrome components: A randomized controlled trial
Source: J Physiol. 2023 Nov 28;602(23):6463–77. doi: 10.1113/JP285366 (PMC11607890; doi:10.1113/JP285366)
Supplement: Supplementary file 2 — Supporting information Baseline and monthly evolution of the 24 h caloric intake and physical activity monitoring in each experimental group [file TJP-602-6463-s002.docx]

**Supporting information**

Baseline and monthly evolution of the 24-hour caloric intake (% macronutrient) and physical activity monitoring (wristband-based) in each experimental group. Data are presented as mean ± SD.

|  |  | | **AMEX (n=42)** | | | |  | **p-value** |
| --- | --- | --- | --- | --- | --- | --- | --- | --- |
|  |  | | **Baseline** | **4 weeks** | **8 weeks** | **16 weeks** |  |  |
| Pre-exercise calorie intake (kcals) |  | 424±34 | | 424±61 | 419±19 | 427±31 |  | 0.34 |
| 24-h calorie intake (kcal/day) |  | 2109±522 | | 2245±289 | 2146±314 | 2090±601 |  | 0.47 |
| % Carbohydrate |  | 50±10 | | 50±7 | 48±6 | 49±8 |  | 0.10 |
| % Fat |  | 35±9 | | 35±7 | 36±5 | 34±6 |  | 0.06 |
| % Saturated fat |  | 38±10 | | 37±7 | 38±6 | 41±7 |  | 0.93 |
| % Protein |  | 12±4 | | 12±6 | 15±6 | 17±3 |  | 0.23 |
| Physical activity (steps/day) |  | 6430±1301 | | 6502±834 | 6416±1131 | 6305±1385 |  | 0.55 |
| Time standing (min/day) |  | 188±119 | | 169±86 | 194±109 | 207±95 |  | 0.09 |
| Time in supine rest (min/day) |  | 500±190 | | 476±176 | 491±201 | 517±190 |  | 0.41 |

|  |  | | **PMEX (n=59)** | | | |  | **p-value** |
| --- | --- | --- | --- | --- | --- | --- | --- | --- |
|  |  | | **Baseline** | **4 weeks** | **8 weeks** | **16 weeks** |  |  |
| Pre-exercise calorie intake (kcals) |  | 828±48 | | 830±30 | 829±28 | 825±40 |  | 0.41 |
| 24-h calorie intake (kcal/day) |  | 2307±445 | | 2319±557 | 2299±591 | 2292±440 |  | 0.76 |
| % Carbohydrate |  | 44±7 | | 48±5 | 48±7 | 50±8 |  | 0.08 |
| % Fat |  | 33±6 | | 31±5 | 33±9 | 29±4 |  | 0.11 |
| % Saturated fat |  | 38±3 | | 38±9 | 36±8 | 38±7 |  | 0.44 |
| % Protein |  | 23±5 | | 21±7 | 18±7 | 21±3 |  | 0.07 |
| Physical activity (steps/day) |  | 5898±1275 | | 6014±1411 | 5988±1332 | 6109±1545 |  | 0.27 |
| Time standing (min/day) |  | 203±113 | | 199±117 | 200±187 | 197±100 |  | 0.51 |
| Time in supine rest (min/day) |  | 524±167 | | 523±201 | 499±192 | 516±156 |  | 0.12 |

|  |  | | **CONTROL (n=38)** | | | |  | **p-value** |
| --- | --- | --- | --- | --- | --- | --- | --- | --- |
|  |  | | **Baseline** | **4 weeks** | **8 weeks** | **16 weeks** |  |  |
| Pre-exercise calorie intake (kcals) |  | ------- | | ------- | ------- | -------- |  |  |
| 24-h calorie intake (kcal/day) |  | 2207±495 | | 2217±437 | 2099±559 | 2073±530 |  | 0.18 |
| % Carbohydrate |  | 46±5 | | 48±6 | 47±9 | 49±7 |  | 0.08 |
| % Fat |  | 35±3 | | 32±5 | 32±6 | 33±4 |  | 0.19 |
| % Saturated fat |  | 40±8 | | 33±6 | 36±8 | 41±6 |  | 0.21 |
| % Protein |  | 19±2 | | 20±5 | 20±9 | 18±3 |  | 0.17 |
| Physical activity (steps/day) |  | 5430± 313 | | 6009±1423 | 6106±1276 | 6005±1457 |  | 0.07 |
| Time standing (min/day) |  | 195±88 | | 199±98 | 187±203 | 207±101 |  | 0.29 |
| Time in supine rest (min/day) |  | 488±201 | | 503±158 | 493±189 | 492±194 |  | 0.52 |
